# Large Scale SARS-CoV-2 Molecular Testing and Genomic Surveillance Reveal Prolonged Infections, Protracted RNA shedding, and Viral Reinfections

**DOI:** 10.3389/fcimb.2022.809407

**Published:** 2022-04-11

**Authors:** C. Paul Morris, Chun Huai Luo, Jaiprasath Sachithanandham, Maggie Li, Matthew Schwartz, David C. Gaston, Victoria Gniazdowski, Nicolas Giraldo-Castillo, Adannaya Amadi, Julie M. Norton, William F. Wright, Eili Y. Klein, Andrew Pekosz, Heba H. Mostafa

**Affiliations:** ^1^ Johns Hopkins School of Medicine, Department of Pathology, Division of Medical Microbiology, Johns Hopkins School of Medicine, Baltimore, MD, United States; ^2^ National Institute of Allergy and Infectious Disease, National Institutes of Health, Bethesda, MD, United States; ^3^ W. Harry Feinstone Department of Molecular Microbiology and Immunology, Johns Hopkins Bloomberg School of Public Health, Baltimore, MD, United States; ^4^ Division of Infectious Diseases, Department of Medicine, Johns Hopkins School of Medicine, Baltimore, MD, United States; ^5^ Department of Emergency Medicine, Johns Hopkins School of Medicine, Baltimore, MD, United States; ^6^ Center for Disease Dynamics, Economics, and Policy, Washington, DC, United States

**Keywords:** SARS-CoV-2, variants, prolonged infection, prolonged shedding, reinfection

## Abstract

Large-scale SARS-CoV-2 molecular testing coupled with whole genome sequencing in the diagnostic laboratories is instrumental for real-time genomic surveillance. The extensive genomic, laboratory, and clinical data provide a valuable resource for understanding cases of reinfection versus prolonged RNA shedding and protracted infections. In this study, data from a total of 22,292 clinical specimens, positive by SARS-CoV-2 molecular diagnosis at Johns Hopkins clinical virology laboratory between March 11^th^ 2020 to September 23^rd^ 2021, were used to identify patients with two or more positive results. A total of 3,650 samples collected from 1,529 patients who had between 2 and 20 positive results were identified in a time frame that extended up to 403 days from the first positive. Cycle threshold values (Ct) were available for 1,622 samples, the median of which was over 30 by 11 days after the first positive. Extended recovery of infectious virus on cell culture was notable for up to 70 days after the first positive in immunocompromised patients. Whole genome sequencing data generated as a part of our SARS-CoV-2 genomic surveillance was available for 1,027 samples from patients that had multiple positive tests. Positive samples collected more than 10 days after initial positive with high quality sequences (coverage >90% and mean depth >100), were more likely to be from unvaccinated, or immunosuppressed patients. Reinfections with viral variants of concern were found in 3 patients more than 130 days from prior infections with a different viral clade. In 75 patients that had 2 or more high quality sequences, the acquisition of more substitutions or deletions was associated with lack of vaccination and longer time between the recovered viruses. Our study highlights the value of integrating genomic, laboratory, and clinical data for understanding the biology of SARS-CoV-2 as well as for setting a precedent for future epidemics and pandemics.

## Introduction

The molecular detection of SARS-CoV-2 has been the gold standard for COVID-19 diagnosis since the beginning of the pandemic. The infrequency of diagnostic tests in March 2020 that limited testing to symptomatic patients under investigation was quickly replaced with large scale screening for both symptomatic and asymptomatic individuals (Anonymous and Centers for Disease Control and Prevention). Although a single positive result is sufficient for making a COVID-19 diagnosis, repeated testing of patients with positive test results has been a notable practice since the beginning of the pandemic. Hospitalized positive patients were re-tested to make infection control related decisions (Gniazdowski et al., 2020). In addition, re-testing is common in immunocompromised patients with symptoms, patients scheduled for certain procedures, as well as patients who develop new symptoms after resolution of COVID-19 (Dong et al., 2021).

Repeat testing revealed that detection of SARS-CoV-2 RNA can be prolonged for up to several months after the start of symptoms (Fontana et al., 2021). The significance of prolonged RNA shedding is dependent on the patient population and the clinical context. In patients with severe disease, the median duration of RNA shedding was longer than mild/asymptomatic cases (Liu et al., 2020; Xu et al., 2020; Zheng et al., 2020). Most cases of prolonged RNA shedding were associated with non-infectious viral recovery. Recovery of infectious virus from patients with extended RNA shedding was mainly reported from immunocompromised patients (Avanzato et al., 2020; Aydillo et al., 2020; Choi et al., 2020; Baang et al., 2021; Tarhini et al., 2021; Truong et al., 2021). A few cases of confirmed reinfections were reported; however, those cases are difficult to differentiate from prolonged infections or RNA shedding without whole genome sequencing. The Centers for Disease Control and Prevention (CDC) has defined the criteria of probable reinfections to either (a) a repeat positive molecular test 90 days after the initial infection regardless of symptoms; or (b) a repeat positive molecular test 45 days after the initial infection in the presence of symptoms consistent with COVID-19 (CDC. Centers for Disease Control and Prevention).

The Johns Hopkins clinical virology Laboratory started molecular testing for SARS-CoV-2 on March 11^th^ 2020, and SARS-CoV-2 whole genome sequencing for understanding the genomic diversity started with the diagnosis of the first positives (Uhteg et al., 2020; Thielen et al., 2021). As of October 9^th^, 2021, the laboratory has tested a total of 555,983 specimens, identified a total of 28,904 positives, and sequenced a total of 8,027 genomes. In this study, we identified patients who had more than one positive result in our laboratory. The time between the first and subsequent tests, cycle threshold (Ct) values, clinical data, and genomic data were examined in addition to cell culture of selected samples to differentiate cases of prolonged RNA shedding, persistent infection, and reinfections. The impact of vaccination and immune suppression on prolonged shedding was evaluated.

## Materials and Methods

### Ethical Considerations and Data Availability

Ethical approval for this study was obtained from the Johns Hopkins Institutional Review Board (IRB00221396) with a waiver of consent. Whole viral genomes were made publicly available at GISAID.

### Data and Sample Selection

Molecular diagnosis for SARS-CoV-2 at Johns Hopkins diagnostic laboratory is performed by different assays that include the NeuMoDx (Qiagen) (Mostafa et al., 2020a; Mostafa et al., 2020b), cobas (Roche) (Mostafa et al., 2020a), Aptima (Hologic), the Xpert Xpress SARS-CoV-2/Flu/RSV (Cepheid) (Mostafa et al., 2020c), the ePlex respiratory pathogen panel 2 (Roche) (Jarrett et al., 2021), the Accula (Hogan et al., 2020), and the RealStar SARS-CoV-2 assays (altona diagnostics) (Uhteg et al., 2020). Testing was performed in accordance with the manufacturer instructions and the Johns Hopkins laboratory’s validated protocols. Patients with more than one positive result were identified *via* the laboratory information system (SOFT). Only Ct values collected from the NeuMoDx assay were used in analysis as the majority of testing is performed using this system (target used, NSP2 gene). Samples with whole genome sequencing data were identified through our surveillance database. Whole genome sequencing and genomic data analysis were performed as we described previously (Morris et al., 2021; Thielen et al., 2021).

### Post Consensus Analysis of Genomes

High quality genomes were defined as genomes with >90% coverage and a mean depth of >100. For analysis of acquired substitutions and deletions, only high-quality genomes were used and genomic areas with poor coverage were excluded. Manual reviews were performed on the acquired substitutions and deletions using the integrated genomic viewer.

### Cell Culture

Aliquots of swab specimens were cultured on Vero-TMPRSS2 cells as previously described for VeroE6 cells (Gniazdowski et al., 2020). Cultures with cytopathic effect were confirmed for the presence of SARS-CoV-2 by reverse transcriptase PCR.

### Clinical Data Analysis

Clinical data for the cohort were retrieved *via* bulk extraction from a data warehouse that contains all encounter-related information from hospital and outpatient visits to any Johns Hopkins Medical Institutions Facilities in addition to manual reviews of electronic medical charts. Codes associated with immunosuppression are listed in **Table S1**.

### Statistical Analysis

Chi squared or Welch’s t-tests were performed to show associations depending on type and number of results evaluated. Linear regression was performed with Scipy and visualized with Seaborn (Waskom, 2021).

## Results

### Repeat Positives From March 11^th^ 2020 to September 23^rd^ 2021

**Figure 1:**
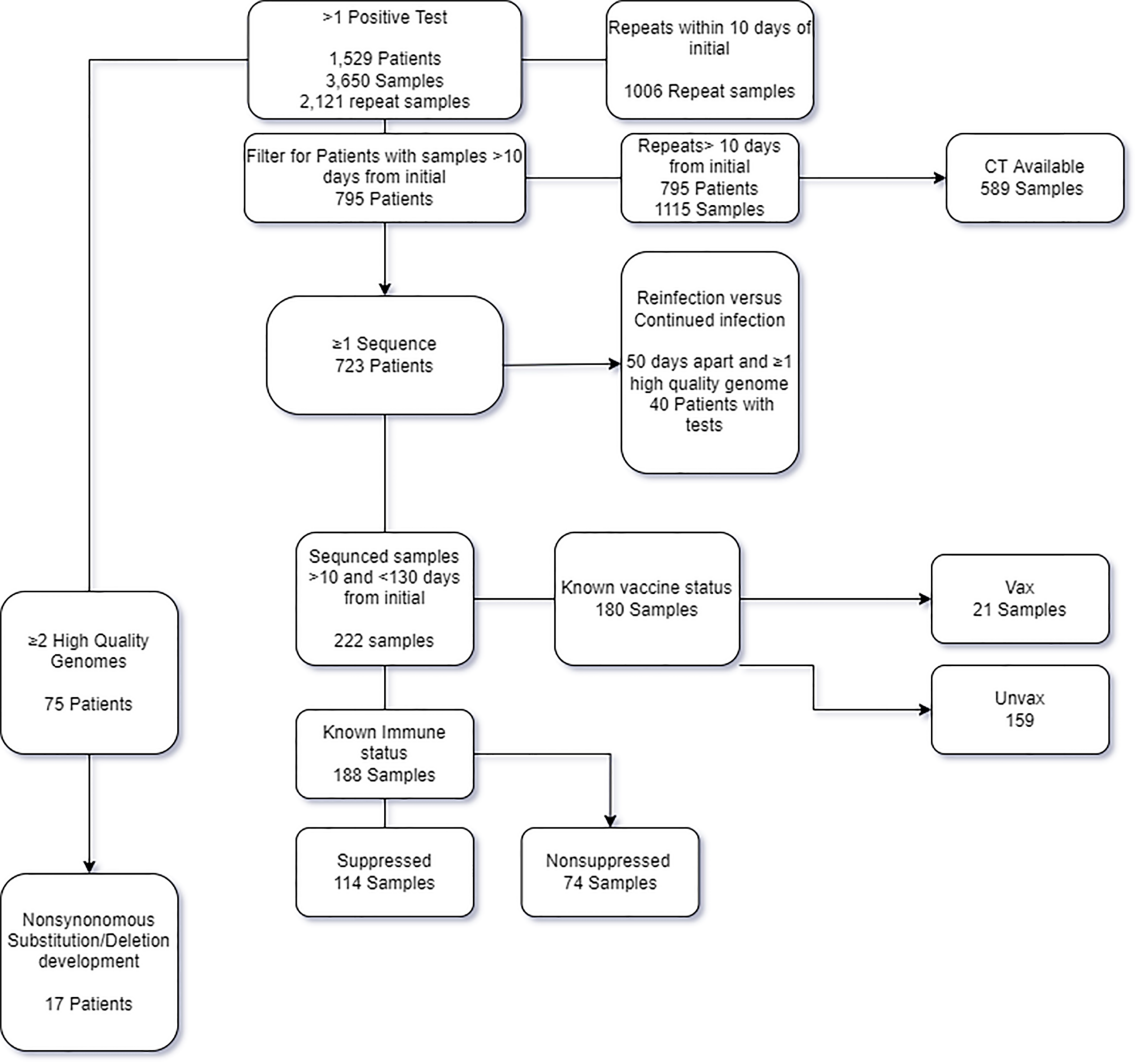
Flow chart of the patients and samples included in the study and analysis.

**Figure 2:**
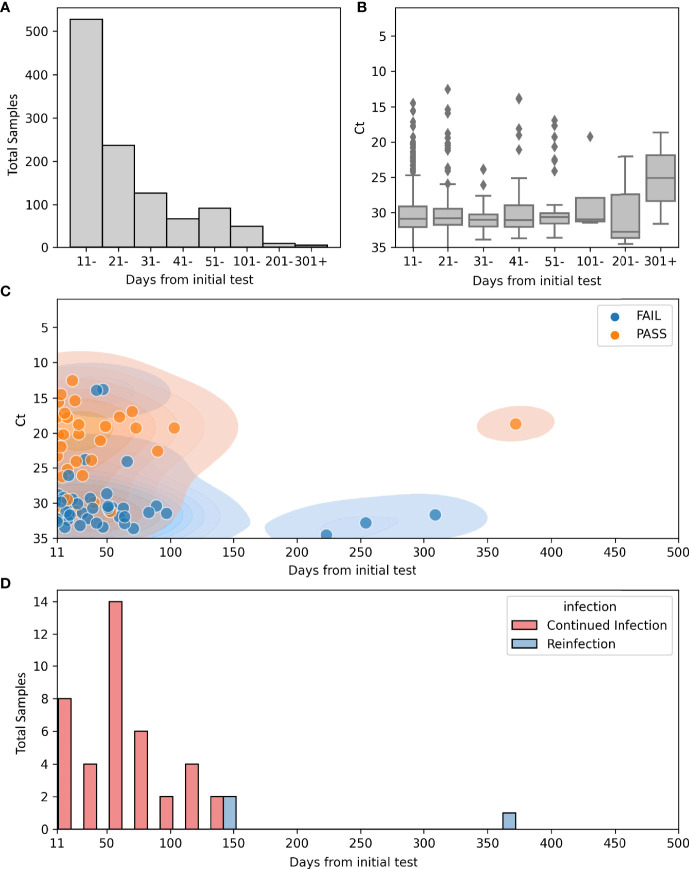
Repeat positives from March 11^th^ 2020 to September 23^rd^ 2021. **(A)** Count plot of total numbers of repeat positives >10 days from the initial positive and the relation to the time after the first positive (N = 1115): 528 (11-20), 237 (21-30), 127 (31-40), 67 (41-50), 92 (51-100), 49 (101- 200), 9 (201-300), 6 (301-400). **(B)** Boxplot for Ct values in repeat positive samples and the relation to the time after the first positive (N = 589): 306 (11-20), 140 (21 -30), 60 (31- 40), 34 (41-50), 40 (51-100), 4 (101-200), 3 (201- 300), 2 (301-400). **(C)** Scatterplot of sequenced repeat samples >10 days from initial positive overlying kernel density estimate (KDE plot) of the same information. Hue indicates quality of sequences. N=222 Total, 155 failed quality metrics, 67 High quality genomes. **(D)** Histogram of days from initial positive sample in continued infection (Red, N = 40) or reinfection (Blue, N = 3).

In the time frame between March 11^th^ 2020 and September 23^rd^ 2021, a total of 542,948 samples were tested at the Johns Hopkins clinical virology laboratory, of which 28,521 tested positive with a total positivity rate of 5.3%. A total of 1,529 patients had more than one SARS-CoV-2 positive result for during this timeframe, for a total of 3,650 samples (7% of the total positives, **Figure 1**). Repeat positive samples were collected between 0 and 403 days from the original positive sample (**Figure 2A**). a total of 943 of the 2,057 repeat samples were tested within the first 10 days of the first positive sample. The majority of the rest were testing that was primarily repeated between 11-20 days (N = 528) after the first positive and only 63 were tested 100 days or more after the first positive (**Figure 2A**).

As SARS-CoV-2 continued RNA positivity during the first 10 days of symptoms is expected (Sethuraman et al., 2020), and to exclude repeat testing that was performed for some patients in the same day, we limited the majority of our analysis to repeat positive samples that occurred more than 10 days from the original positive. Ct values were available for 589 of the 1,115 repeat samples > 10 days from the initial positive result (52%; **Figures 2B, C**). Although Ct values were lower prior to 11 days from the initial sample (data not shown), the majority of samples collected after 11 days from the first positive showed CT values greater than 30 (**Figures 2B, C**). The majority of samples were collected prior to 100 days, and only two Ct values were available for samples collected 300 days after the first positive (**Figure 2B**).

Of the 1,151 repeat samples more than 10 days from the initial positive, whole genome sequencing was attempted on 222 samples collected from 173 of the 795 patients from this cohort (**Figure 2C**). Of the sequenced samples in this cohort, 69.8% did not meet the quality cut-off scores (155 of 222) (**Table 1**). The mean Ct values of samples with high quality sequences was 22.1 in contrast to a mean Ct of 30.4 for those with low quality (data detailed in **Table S2** and summarized in **Table 1**).

**Table 1:** Samples with whole genome sequencing data in our cohort of repeats collected 11 days or more after the first positive.

Days after the first positive	Genomes with high quality			Genomes with low quality			p value
	Number	Average Ct	Stdev	Number	Average Ct	Stdev	
11- 20	28	21.1	5.7	43	31.2	2.3	0.00006
21-50	25	21.1	5.7	46	30.4	6.5	0.02
51+	14	19.3	4.9	66	31.4	2.3	0.0009
total	67	20.24	5.4	155	31.24	4.2	1.00E-09

High quality genomes were defined as sequences with coverage >90% and a mean depth of >100. Ct, cycle threshold; stdev, standard deviation.

### Prolonged Shedding Versus Reinfections

To differentiate cases of prolonged shedding from reinfections based on viral genomics analyses, genomes from the same patients were compared. For this analysis, we focused primarily on the 40 patients in our cohort who had at least two positive SARS-CoV-2 PCR tests >50 days apart and with at least one high quality genome (**Table S3**). Patients from this group tested positive between 2 and 16 times and the time between the first and last positive tests was between 51 and 372 days. In total, there were 162 positive samples of which 109 were sequenced and all available genomes were analyzed regardless of time from initial positive. Despite low quality sequences of genomes from many of the samples collected at later time points, 14 of these patients showed evidence of prolonged shedding of RNA, consistent with the initial infecting virus, and only 3 showed genomic evidence of reinfection (**Table 2** and **Table S3**). Two were initially infected with one clade, [20G or 20I (Alpha)], followed by a later infection with the Delta variant. The third patient was considered a reinfection because the second infection was with the Delta variant which was not circulating when the patient first tested positive 372 days earlier (**Table 2**). The earliest positive test from a reinfection occurred 133 days following the initial infection (**Figure 2D**), and Continued RNA detection was noted as far as 139 days following the initial positive.

**Table 2:** Prolonged shedding, persistent infections, versus reinfections.

PID	Vaccinated	Immunosuppressed	Days from the first positive	HPID	Cell culture	Ct	% Coverage	Depth	Lineage	Clade
**Reinfections**										
1	Yes	No	0							
			372	HP08786		18.7	98.68	400.00	B.1.617.2	21A (Delta)
13	Yes	Yes	0	HP05124			99.60	400.00	B.1.2	20G
			11	HP04658		17.73	98.60	385.09	B.1.2	20G
			144	HP08875			86.10	172.40	AY.7.1	21A (Delta)
28	Yes	No	0	HP05001			98.87	333.78	B.1.1.7	20I (Alpha, V1)
			133	HP09101			95.54	381.78	B.1.617.2	21A (Delta)
**Persistent infection**										
10	No	Yes	0			24.19				
			27			23.65				
			33	HP13078	Positive	23.83	0.00	3.00	None	Attempted
			38	HP13068	Negative	23.93	97.25	349.00	A.3	19B
			53		Positive	20.69				
			81	HP13137	Negative		95.50	378.49	A.3	19B
12	No	Yes	0							
			6	HP13142	Positive	16.99	99.60	400.00	B.1	20C
			42	HP13143	Positive	13.93	2.69	9.15	None	Attempted
			46							
			60	HP13074	Positive	17.71	98.73	400.00	B.1	20C
			73	HP13075	Negative	19.28	98.75	400.00	B.1	20C
			84			22.47				
16	No	Yes	0	HP13076	Positive	16.5	99.01	400.00	B.1.520	20C
			39	HP13144	Positive		99.59	400.00	B.1.520	20C
			65							
			70	HP13077	Positive	16.95	99.57	400.00	B.1	20C
**Prolonged shedding**										
15	No	Yes	0	HP12091	Positive	18.62	0.00	400.00	B.1.1.434	20B
			20							
			26	HP12092	Positive	24.1	0.00	378.28	B.1.1.434	20B
			33	HP12093	Negative			235.88	B.1.1.434	20B
			38	HP12094	Negative			400.00	B.1.1.434	20B
			103	HP02090		19.27	99.57	400.00	B.1.1.434	20B
			117	HP02480			98.59	142.00	B.1.1.434	20B
			126	HP02621			64.60	63.50	None	20B
			132	HP03033			32.65	23.28	None	20B
			139	HP03236			9.04	5.85	None	Attempted
19	Yes	Yes	0	HP02155		13.69	99.60	400.00	B.1.409	20A
			19							
			57	HP01654			99.59	400.00	B.1.409	20A
			61	HP01599			99.59	400.00	B.1.409	20A
			77	HP02479			13.62	9.00	None	Attempted
20	No	Yes	0			19.15				
			1	HP03788		14.16	99.60	400.00	B.1.526	21F (Iota)
			7			15.31				
			14	HP04418		14.52	98.60	400.00	B.1.526	21F (Iota)
			19	HP04748		17.78	99.60	400.00	B.1.526	21F (Iota)
			21	HP04804			98.60	400.00	B.1.526	21F (Iota)
			25	HP04922		15.41	99.60	400.00	B.1.526	21F (Iota)
			28	HP05155		18.78	97.75	400.00	B.1.526	21F (Iota)
			31	HP05251		26.11	97.89	354.79	B.1.526	21F (Iota)
			35							
			39							
			64	HP06212		32.91	56.72	172.78	None	21F (Iota)
3	No		0	HP13038	Positive	20.22	99.60	400.00	B.1	20A
			50			25.13				
			50	HP10152			87.25	165.00	B.1	20A
			54	HP13039	Negative	30.61	89.76	102.34	B.1	20A
4	No	Yes	0	HP13053	Positive	21.41	99.60	400.00	B.1.494	20C
			36			29.45				
			37							
			64	HP13054	Negative		76.25	40.85	B.1.446	20C
			124			31.53				
11	No	No	0	HP13040	Negative	25.43	99.60	400.00	B.1.369	20C
			38			31.51				
			53	HP13041	Negative	31.21	90.19	227.39	B.1.369	20C
27	No	No	0	HP13044	Positive	14.47	99.60	400.00	B.1.369	20C
			25			33.14				
			51	HP13045	Negative	30.73	67.59	227.37	None	20C
29	Yes	Yes	0	HP02004			99.60	400.00	B.1.2	20G
			3	HP02005		15.53	99.60	400.00	B.1.2	20G
			12	HP02006			99.60	387.00	B.1.2	20G
			17	HP02007			98.60	400.00	B.1.2	20G
			23							
			24			20.48				
			30			21.19				
			37	HP02008		29.34	37.73	26.00	None	20C
			44	HP02009			81.92	117.00	B.1.2	20G
			51	HP02010		30.48	4.47	4.00	None	Attempted
34	Yes	No	0	HP13046	Negative	29.6	99.60	400.00	B.1	20C
			92	HP13047	Negative		46.98	52.01	None	20C
35	Yes	No	0	HP13036	Negative	32.86	99.59	212.41	B.1.110	20A
			16			29.14				
			57	HP13037	Negative		87.83	64.10	B.1	20A
37	Yes	Yes	0	HP02713	Positive		99.60	400.00	B.1.2	20G
			23	HP03492			99.60	368.98	B.1.2	20G
			38							
			39							
			45	HP04440		21.11	98.60	387.16	B.1.2	20G
			50	HP04626		28.69	31.43	18.08	None	20G
			57	HP04882		29.1	94.15	314.68	B.1.2	20G

PID, patient identifier; HPID, identification of samples with whole genome sequencing; Ct, cycle threshold.

Of the 14 patients with genomic evidence of prolonged shedding, Cell culture was attempted for 28 samples from this cohort to assess prolonged active infection versus prolonged RNA shedding. Three immunocompromised patients showed prolonged recovery of infectious virus on cell culture (**Table 2**, patients 10, 12, and 16) for 53, 60, and 70 days after the first positive, indicating persistent infection. Notably, three additional immunocompromised patients had prolonged shedding (patients 15, 19, and 20, **Table 2**) with a notable low Ct values or recovery of complete genomes for up to 117, 61, and 31 days after the first positive. The rest of the 14 patients from this cohort had extended shedding at higher Ct values, and 6 patients had negative cell culture results suggesting protracted RNA shedding rather than persistent infection (**Table 2**).

### Impact of Vaccination on Prolonged Viral Shedding

**Figure 3:**
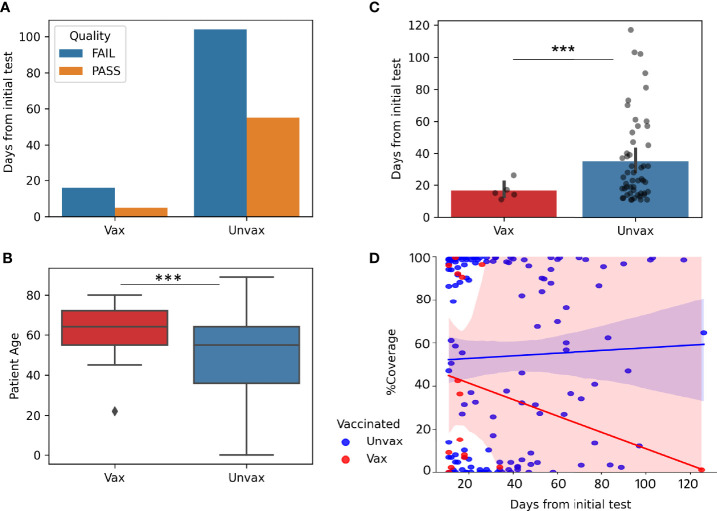
Impact of Vaccination on prolonged viral shedding. Genomes from repeat positive samples within the first 130 days after the initial positives (N = 21 Vax “vaccinated” and 159 Unvax “unvaccinated”). **(A)** Quality of genomes in samples from vaccinated and unvaccinated patients. **(B)** Boxplot of Patient Age in samples from vaccinated (Median 64 years) and unvaccinated (median 55 years). **(C)** Barplots with overlayed strip plots of the recovery of high-quality genomes compared in relation to days after the first positive. **(D)** Implots of genome coverage compared to days from the initial positive. ***p < 0.0001.

To study the impact of vaccination on prolonged viral shedding, we compared the genomic data of samples from vaccinated compared to unvaccinated patients. We limited this analysis to sequenced samples that were positive 11 to 130 days from the initial positive test (in order to remove possible repeat infections, as repeat infections started to be seen at 130 days). Patients who had received the full vaccination series at least 2 weeks prior to the first positive test were classified as vaccinated in this cohort. Only 21 samples from vaccinated patients with repeated positive tests met these criteria (**Figure 3A**) compared to 159 repeat positive samples from unvaccinated patients. Only 5 of 21(23%) genomes within this timeframe met our quality scores in vaccinated patients compared to 55 of 159(35%) in unvaccinated patients, despite older median age in the vaccinated patients (64 compared to 55, p=0.00065) (**Figure 3B**), and lower median days from the initial test for all samples where sequencing was attempted (17 days vaccinated, 32 days unvaccinated, p=0.0003, **Figure 3C**).In general, genomes of repeat positives from vaccinated patients after 20 days from the first positives were very few which caused a notable trend of a decrease in coverage with time when compared to the unvaccinated group (**Figure 3D**).

### Impact of Immunosuppression on Prolonged Viral Shedding

**Figure 4:**
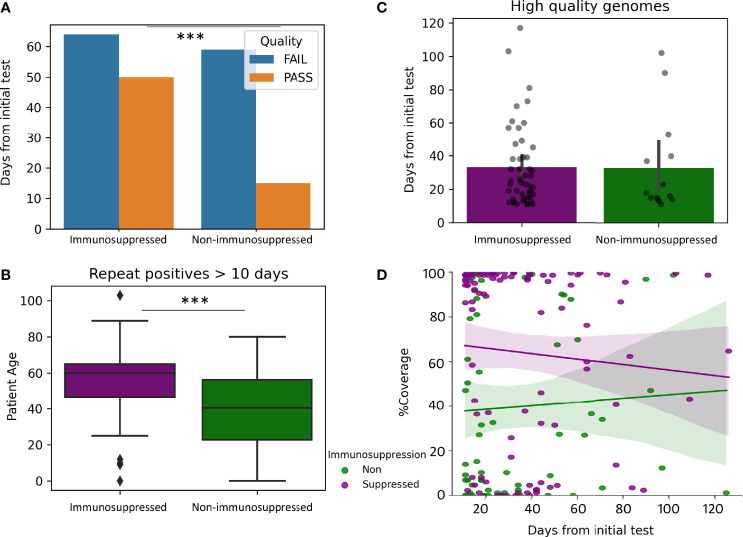
Impact of Immunosuppression on prolonged viral shedding. Genomes from repeat positive samples within the first 130 days after the initial positives (N = 114 immunosuppressed and 74 non-immunosuppressed). **(A)** Quality of genomes in samples from immunosuppressed and non-immunosuppressed patients. **(B)** Boxplot of patient age in samples from immunosuppressed (Median 60 years) and non-immunosuppressed (median 40.5 years). **(C)** Barplots with overlayed strip plots of the recovery of high-quality genomes compared in relation to days after the first positive. **(D)** Implots of genome coverage compared to days from the initial positive in immunosuppressed and non-immunosuppressed patients. ***p < 0.00001.

To evaluate the impact of immunosuppression the coverage of the SARS-CoV-2 genome in 74 samples from patients without immunosuppression were compared to 114 samples from patients with immunosuppression (greater than 10 days and less than 130 days from the initial positive). High quality genomes were more represented at a higher percentage in Immunosuppressed compared to non-immunosuppressed patients (43% compared to 20%, p-value 0.0009, **Figure 4A**). Immunosuppressed patients were older than non-immunosuppressed patients (median years 60 compared to 40.5, p-value >0.0001, **Figure 4B**). However, high quality genomes were recovered for similar mean days between immunosuppressed and non-immunosuppressed patients (33.1, compared to 31.5 days, **Figure 4C**) with similar coverage over-time (**Figure 4D**). Additional analysis was performed to see how vaccination impacted Immunosuppressed patients. Of the sampled from immunosuppressed patients, 13 were from vaccinated and 94 were from unvaccinated patients. The percentages of samples that passed quality control from vaccinated and unvaccinated immunosuppressed were similar, but high-quality genomes were obtained for shorter periods of time in samples from vaccinated compared to unvaccinated patients (16.6 days compared to 35.7 days, p=0.009) (data not shown).

### Prolonged Viral Shedding and Genomic Changes

**Figure 5:**
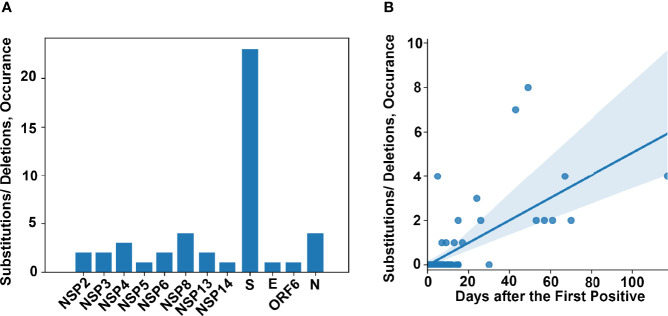
Genomic changes in prolonged viral shedding. **(A)** Frequency of amino acid changes (substitutions and deletions) per protein in a cohort of 75 patients with two or more complete genomes from samples collected 0 - 117 days after the first positive. **(B)** Amino acid changes as a factor of time between collected positive samples.

In order to study the development of new substitutions or deletions within the genome of SARS-CoV-2 over-time, within the same patient, we identified all patients for whom we had at least 2 high quality genomes from different samples and whose genomic data was consistent with prolonged shedding or persistent infection but not reinfection. This analysis was not limited to a specific timeframe between positive samples. We identified 75 patients that met these criteria. The timeframe between the initial and subsequent high-quality genomes was between 0 and 117 days (detailed in **Table S4**). A total of 48 amino acid (AA) substitutions or deletions developed in 17 of these patients (**Table 3**). Of the 17 patients that acquired substitutions or deletions, 16 were unvaccinated and 14 were immunosuppressed. The substitutions and deletions which developed and the number of instances in our cohort can be seen in **Table S5**, along with information on how many times these specific substitutions or deletions have been seen among all the genomes sequenced in our laboratory and lineages associated with these changes in multiple instances. Of the 48 substitutions or deletions noted, three spike protein deletions (L141del, G142del, and V143del) and one spike protein substitution (E484K) occurred in two instances. Each of these substitutions or deletions that occurred more than once in this dataset was a part of disparate lineages, especially S:E484K which was seen in at least 19 lineages by our group (**Table S5**). The most common protein to develop substitutions or deletions was the Spike protein (**Figure 5A**) A correlation between AA changes and days between recoverable genomes was noted (**Figure 5B**, p < 0.00001). Recoverable genomes over 24 days from the original positive showed a minimum of two non-synonymous substitutions or deletions (**Table 3**).

**Table 3:** Prolonged viral shedding and genomic changes.

PID	Positives	Sequenced	HQ Genomes	Days between first and last positives	Days between first and last HQ Genomes	First HQ genome lineage	last HQ genome lineage	Number Acquired subs/dels	Acquired AA substitutions/deletions	Vaccinated	Immunosuppressed	Monoclonal antibody	Convalescent Plasma
PIDVWVGDCM	2	2	2	5	5	B.1.2	B.1.2	4	S:G142del,S:V143del,S:Y144del,S:L141del	No	Yes		Yes
PIDWUGBIZH	5	4	3	14	7	B.1.637	B.1.637	1	S:H146Q	No	Yes		Yes
PIDCXIYIPP	6	5	4	20	9	B.1.243	B.1.243	1	NSP8:F6L	No	Yes		Yes
PIDLUEEQYV	5	4	2	13	13	B.1.1.7	B.1.1.7	1	NSP14:D126Y	No	Yes		
PIDEAUSZWF	2	2	2	15	15	B.1.2	B.1.2	2	NSP2:E490K,S:E484K	No			
PIDRSHNQNS	12	8	5	51	17	B.1.2	B.1.2	1	E:V70F	No	Yes		Yes
PIDGBIBYYQ	7	4	4	24	24	Q.4	Q.4	3	NSP4:H313Y,N:F307L,S:R102G	No	Yes		Yes
PIDBXQZZLV	5	4	4	26	26	B.1.526	B.1.526	2	S:G446V,S:E406Q	Yes	Yes		Yes
PIDLCYVIUV	2	2	2	37	37	B.1.564	B.1	2	S:L270F,NSP2:T85I	No			
PIDFTUQFAV	7	3	2	81	43	A.3	A.3	7	S:R190S,S:L244del,NSP4:F317S,S:T1006I,NSP3:F1646I,NSP4:G232V,S:A243del	No	Yes		
PIDSDHALQC	5	4	4	49	49	B.1.637	B.1.637	8	S:T95I,S:G142del,NSP13:T115K,S:V143del,S:L141del,S:E484Q,NSP8:T123I,S:W152R	No	Yes		
PIDHCPHTBW	3	2	2	53	53	B.1.369	B.1.369	2	NSP5:Q192stop,N:T271I	No			
PIDXTTUNJN	9	5	4	57	57	B.1.2	B.1.2	2	S:E484K,NSP6:L37F	No	Yes	Yes	
PIDNXQFFZG	6	4	3	77	61	B.1.409	B.1.409	2	NSP8:I156L,NS6:E13K	No	Yes	Yes	Yes
PIDHNGFDXK	7	4	3	84	67	B.1	B.1	4	NSP8:T148I,S:P1079S,NSP3:T820I,N:P326L	No	Yes		
PIDLYROPCD	4	3	3	70	70	B.1.520	B.1	2	NSP6:M86I,N:T205I	No	Yes		
PIDLVBEHYS	11	9	6	139	117	B.1.1.434	B.1.1.434	4	NSP2:K534R,S:N334K,NSP13:D56G,S:S939F	No	Yes	Yes	

PID, patient identifier; HPID, identification of samples with whole genome sequencing; HQ, High quality genomes were defined as sequences with coverage >90% and a mean depth of >100. Subs/dels, substitutions/deletions; AA, amino acid.

**Figure 6:**
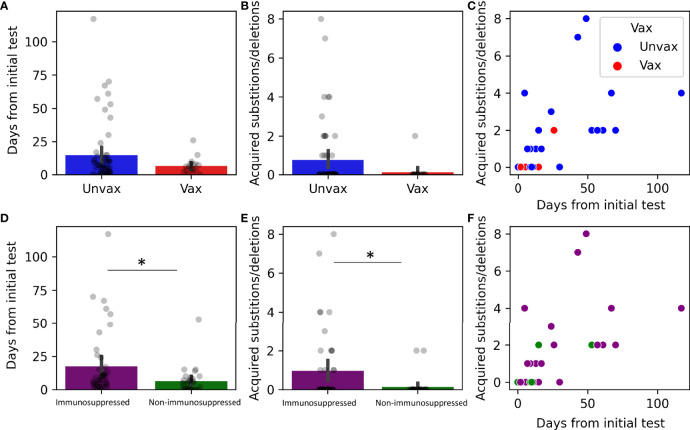
Genomic changes in prolonged viral shedding by vaccination and immune status. Substitutions and deletions in 75 patients by status: vaccinated (N =17, **A–C**) and immunosuppressed (N=44, **D–F**). **(A, D)** Barplots with overlayed strip plots of mean time of prolonged shedding of complete genomes in each group, **(B, E)** Barplots with overlayed strip plots of amino acid change frequency in each group, **(C, F)** Scatterplots of the correlation of amino acid change and the days after the first positive in each group. *p < 0.05. Unvax, unvaccinated; Vax, vaccinated.

We next examined the impact of vaccination and immune suppression on the accumulation of nonsynonymous mutations. Of the 75 patients that had multiple recoverable genomes, 17 were vaccinated (**Figures 6A**–**C**). Only one vaccinated patient developed nonsynonymous mutations (**Table 3**). Although vaccinated patients that had multiple recoverable genomes showed a lower mean time of recoverable genomes, and a lower mean number of developed mutations, this did not reach significance (**Figures 6A**–**C**). Immunocompromised status was associated with longer periods between the recoverable genomes (**Figure 6D**, p=0.022) and a higher number of substitutions or deletions (**Figures 6E, F**, p=0.022).

## Discussion

In this study, we combined large scale laboratory diagnostic data with whole genome sequencing data for surveillance to analyze prolonged SARS-CoV-2 shedding and reinfections. Clinical data analysis was performed to study the impact of vaccination and immunocompromised status on genomic changes over-time. Cell culture was performed in certain cases with extended viral shedding combined with low Ct values to characterize cases of prolonged active infection. Our data showed that the majority of patients with prolonged positive SARS-CoV-2 molecular tests have their subsequent positives collected within the first 50 days of the initial positive with Ct values that are likely higher than 30. Prolonged shedding after 50 days was largely associated with low quality genomes reflecting low viral loads except in 17 patients, 14 of whom had subsequent genomes matching the initial, indicating prolonged shedding and 3 had a second genome of a different clade, indicating reinfection. Vaccination reduced the likelihood of the recovery of good quality genomes but immunocompromised status contributed to the increased duration of viral RNA shedding and the accumulation of genomic changes. The spike gene was the region of the genome most prone to nonsynonymous changes in our cohort, but overall, the development of nonsynonymous mutations and AA changes was infrequent.

Prolonged SARS-CoV-2 shedding was shown to extend to multiple months after the onset of symptoms (Fontana et al., 2021). Prolonged shedding though might not correlate with prolonged infectiousness or recovery of viable virus. Previously we showed that shedding of infectious virus can extend to more than 3 weeks after the initial positive (Gniazdowski et al., 2020). In this study, we show the recovery of infectious virus for up to 70 days after the first positive in a subset of immunocompromised patients. This data is consistent with previous reports that showed prolonged SARS-CoV-2 replication and infectiousness in immunocompromised patients (Aydillo et al., 2020; Baang et al., 2021; Tarhini et al., 2021). In general, the recovery of infectious virus correlated with lower Ct values consistent with higher viral loads (Basile et al., 2020; Bullard et al., 2020; Gniazdowski et al., 2020). The value of differentiating prolonged shedding from prolonged infection include controlling the transmission of infection in patients with actively replicating virus as well as optimizing antiviral intervention strategies. Our data is consistent with previous reports that show that prolonged active infection is remarkably less frequent than prolonged shedding of viral RNA and are primarily associated with immunocompromised patient populations.

Reinfection with SARS-CoV-2 was previously reported and a time frame of 45 days was proposed to suggest possible reinfections when a positive test is associated with symptoms consistent with COVID-19. Genomic sequencing is the only method that can conclusively characterize reinfection cases when the initial genomes are compared to subsequent positives. When initial isolates are not available, it might be possible to characterize reinfections if the lineage identified is an emerging variant that was not circulating when the patient was previously diagnosed. Interestingly, in the time frame when the Delta variant became predominant (Morris et al., 2021), we only identified three patients who had a previous positive in our institution. Even though this data might be largely impacted by the availability of other testing sites outside of the Johns Hopkins system, within the population of patients tested in our laboratory, we identified breakthrough cases after vaccination in rates that were much higher than reinfections (Luo et al., 2021).

The evolution of SARS-CoV-2 variants associated with increased transmissibility or escape from vaccine induced or natural immune responses has been globally concerning. Specific variants showed significant reduction in neutralization by monoclonal antibodies and convalescent plasma (Hoffmann et al., 2021; Planas et al., 2021). Those variants attracted the attention to specific changes within SARS-CoV-2 genome that could be of therapeutic concern. Certain spike changes were shown to impact the action of monoclonal antibodies and convalescent sera including L452R, E484K, K417N, and K417T. In our cohort, the spike changes that we observed more than once included the E484K and the deletions 141-143. The E484K in particular was previously reported to reduce the neutralization efficacy and was identified as an escape change the could develop after treatment with certain monoclonal antibodies or convalescent plasma (Baum et al., 2020; Weisblum et al., 2020; Greaney et al., 2021). The deletions 141-144 are within the NTD and were reported to reduce binding to the monoclonal antibody 4A8 (recovered from the convalescent plasma of patients with COVID-19) as do deletions 243-244 (which occurred once in our cohort) (McCarthy et al., 2021). Other changes we detected in our cohort are within epitopes or associated with escape from convalescent sera including W152R, F157S, and G446V (Liu et al., 2021; Suryadevara et al., 2021). Although 9 of the 17 patients in our cohort whom infecting virus developed nonsynonymous mutations received monoclonal or convalescent sera treatments (**Table 3**), associations between those treatments and the rate of developing mutations were not performed due to the incompleteness of this data in some of the clinical charts. In our cohort, the accumulation of genomic changes associated with the prolonged shedding time that was most remarkable in immunosuppressed individuals.

A main finding in our study was the correlation between vaccination and a lower likelihood of prolonged shedding or recovery of good quality genomes after the first 20 days of the initial positive result. Even though, vaccine breakthrough infections correlated with a bias to genomes carrying the S: E484K (Feder et al., 2021; Mostafa et al., 2021), a limited infectious virus shedding was notable when samples from patients were longitudinally analyzed (Ke et al., 2021). SARS-CoV-2 genomic diversity was also shown to decline after widespread vaccination (Niesen et al., 2021). The selected cohort for our analysis was restricted to individuals who had an initial positive when fully vaccinated. This data indicate that vaccination is interrupting the extended prolonged shedding observed since the start of the pandemic with unvaccinated individuals. Our data also show that vaccinated individuals are less likely to accumulate significant genomic changes over time.

In summary, the COVID-19 pandemic challenged the diagnostic laboratories to not only ramp up testing, but to also assist with the nationwide genomic surveillance. The diagnostic laboratories are capable of providing real-time epidemiological and clinical data that are essential for a better understanding of the biology of SARS-CoV-2. Diagnostic laboratories with limited resources can also assist by coordinating sharing real-time positive samples with public health laboratories for surveillance. The workflow of diagnosis, surveillance, and basic research we established at Johns Hopkins laboratory for characterizing SARS-CoV-2 provides a template for other evolving pathogens of concern and emphasizes the power of generating real-time data amid a quickly evolving pandemic.

## Data Availability Statement

The datasets presented in this study can be found in online repositories. The names of the repository/repositories and accession number(s) can be found in the article/**Supplementary Material**.

## Ethics Statement

The studies involving human participants were reviewed and approved by the Johns Hopkins Institutional Review Board (IRB00221396) with a waiver of consent. Written informed consent from the participants’ legal guardian/next of kin was not required to participate in this study in accordance with the national legislation and the institutional requirements.

## Author Contributions

CM data collection and analysis, data interpretation, figures, writing. CL, JS and ML cell culture. DG, VG, NG, AA, JN, MS, and WW. data collection. EK. Clinical data collection and analysis. AP. cell culture and scientific revision. HM study design, data collection and analysis, data interpretation, writing, and fund acquisition. All authors contributed to the article and approved the submitted version.

## Funding

This study was funded by the Centers for Disease Control (contract 75D30121C11061), Johns Hopkins University President’s Fund Research Response, the Johns Hopkins Department of Pathology, the Maryland Department of Health, and the Johns Hopkins Center of Excellence in Influenza Research and Surveillance (HHSN272201400007C). HM is supported by the HIV Prevention Trials Network (HPTN) sponsored by the National Institute of Allergy and Infectious Diseases (NIAID), National Institute on Drug Abuse, National Institute of Mental Health, and Office of AIDS Research of the NIH, DHHS (UM1 AI068613), the NIH RADx-Tech program (3U54HL143541-02S2), and National Institute of Health RADx-UP initiative (Grant R01 DA045556-04S1). The views expressed in this manuscript are those of the authors and do not necessarily represent the views of the National Institute of Biomedical Imaging and Bioengineering; the National Heart, Lung, and Blood Institute; the National Institutes of Health, or the U.S. Department of Health and Human Services.

## Conflict of Interest

The authors declare that the research was conducted in the absence of any commercial or financial relationships that could be construed as a potential conflict of interest.

## Publisher’s Note

All claims expressed in this article are solely those of the authors and do not necessarily represent those of their affiliated organizations, or those of the publisher, the editors and the reviewers. Any product that may be evaluated in this article, or claim that may be made by its manufacturer, is not guaranteed or endorsed by the publisher.

## References

[B1] Anonymous Centers for Disease Control and Prevention Overview of Testing for SARS-CoV-2 (COVID-19). Available at: https://wwwcdcgov/coronavirus/2019-ncov/hcp/testing-overviewhtml. (Updates as of August 2, 2021).

[B8] Avanzato V. A. Matson M. J. Seifert S. N. Pryce R. Williamson B. N. Anzick S. L.. (2020). Case Study: Prolonged Infectious SARS-CoV-2 Shedding From an Asymptomatic Immunocompromised Individual With Cancer. Cell 183, 1901–1912.e9. doi: 10.1016/j.cell.2020.10.049 33248470 PMC7640888

[B10] Aydillo T. Gonzalez-Reiche A. S. Aslam S. van de Guchte A. Khan Z. Obla A.. (2020). Shedding of Viable SARS-CoV-2 After Immunosuppressive Therapy for Cancer. N. Engl. J. Med. 383, 2586–2588. doi: 10.1056/NEJMc2031670 33259154 PMC7722690

[B12] Baang J. H. Smith C. Mirabelli C. Valesano A. L. Manthei D. M. Bachman M. A.. (2021). Prolonged Severe Acute Respiratory Syndrome Coronavirus 2 Replication in an Immunocompromised Patient. J. Infect. Dis. 223, 23–27. doi: 10.1093/infdis/jiaa666 33089317 PMC7797758

[B26] Basile K. McPhie K. Carter I. Alderson S. Rahman H. Donovan L.. (2020). Cell-Based Culture of SARS-CoV-2 Informs Infectivity and Safe De-Isolation Assessments During COVID-19. Clin. Infect. Dis. 73 (9), e2952–e2959. doi: 10.1093/cid/ciaa1579 PMC7665383 33098412

[B32] Baum A. Fulton B. O. Wloga E. Copin R. Pascal K. E. Russo V.. (2020). Antibody Cocktail to SARS-CoV-2 Spike Protein Prevents Rapid Mutational Escape Seen With Individual Antibodies. Science 369, 1014–1018. doi: 10.1126/science.abd0831 32540904 PMC7299283

[B25] Bullard J. Dust K. Funk D. Strong J. E. Alexander D. Garnett L.. (2020). Predicting Infectious Severe Acute Respiratory Syndrome Coronavirus 2 From Diagnostic Samples. Clin. Infect. Dis. 71, 2663–2666. doi: 10.1093/cid/ciaa638 32442256 PMC7314198

[B14] CDC. Centers for Disease Control and Prevention Investigative Criteria for Suspected Cases of SARS-CoV-2 Reinfection (ICR). Available at: https://www.cdc.gov/coronavirus/2019-ncov/php/invest-criteria.html (Accessed October 29, 2020).

[B9] Choi B. Choudhary M. C. Regan J. Sparks J. A. Padera R. F. Qiu X.. (2020). Persistence and Evolution of SARS-CoV-2 in an Immunocompromised Host. N. Engl. J. Med. 383, 2291–2293. doi: 10.1056/NEJMc2031364 33176080 PMC7673303

[B3] Dong X. Zhou Y. Shu X. O. Bernstam E. V. Stern R. Aronoff D. M.. (2021). Comprehensive Characterization of COVID-19 Patients With Repeatedly Positive SARS-CoV-2 Tests Using a Large U.S. Electronic Health Record Database. Microbiol. Spectr. 9, e0032721. doi: 10.1128/Spectrum.00327-21 34406805 PMC8552669

[B36] Feder K. A. Patel A. Vepachedu V. R. Dominguez C. Keller E. N. Klein L.. (2021). Association of E484K Spike Protein Mutation With SARS-CoV-2 Infection in Vaccinated Persons—Maryland, January - May 2021. Clin. Infect. Dis. ciab762. doi: 10.1093/cid/ciab762 34473242 PMC8522365

[B4] Fontana L. M. Villamagna A. H. Sikka M. K. McGregor J. C. (2021). Understanding Viral Shedding of Severe Acute Respiratory Coronavirus Virus 2 (SARS-CoV-2): Review of Current Literature. Infect. Control Hosp. Epidemiol. 42, 659–668. doi: 10.1017/ice.2020.1273 33077007 PMC7691645

[B2] Gniazdowski V. Morris C. P. Wohl S. Mehoke T. Ramakrishnan S. Thielen P.. (2020). Repeat COVID-19 Molecular Testing: Correlation of SARS-CoV-2 Culture With Molecular Assays and Cycle Thresholds. Clin. Infect. Dis. 73 (4), e860–e869. doi: 10.1093/cid/ciaa1616 PMC7665437 33104776

[B30] Greaney A. J. Loes A. N. Crawford K. H. D. Starr T. N. Malone K. D. Chu H. Y.. (2021). Comprehensive Mapping of Mutations in the SARS-CoV-2 Receptor-Binding Domain That Affect Recognition by Polyclonal Human Plasma Antibodies. Cell Host Microbe 29, 463–476.e6. doi: 10.1016/j.chom.2021.02.003 33592168 PMC7869748

[B29] Hoffmann M. Hofmann-Winkler H. Kruger N. Kempf A. Nehlmeier I. Graichen L.. (2021). SARS-CoV-2 Variant B.1.617 Is Resistant to Bamlanivimab and Evades Antibodies Induced by Infection and Vaccination. Cell Rep. 36, 109415. doi: 10.1016/j.celrep.2021.109415 34270919 PMC8238662

[B21] Hogan C. A. Garamani N. Lee A. S. Tung J. K. Sahoo M. K. Huang C.. (2020). Comparison of the Accula SARS-CoV-2 Test With a Laboratory-Developed Assay for Detection of SARS-CoV-2 RNA in Clinical Nasopharyngeal Specimens. J. Clin. Microbiol. 58 (8), e01072–20. doi: 10.1128/JCM.01072-20 PMC7383558 32461285

[B20] Jarrett J. Uhteg K. Forman M. S. Hanlon A. Vargas C. Carroll K. C.. (2021). Clinical Performance of the GenMark Dx Eplex Respiratory Pathogen Panels for Upper and Lower Respiratory Tract Infections. J. Clin. Virol. 135, 104737. doi: 10.1016/j.jcv.2021.104737 33497932

[B38] Ke R. Martinez P. P. Smith R. L. Gibson L. L. Achenbach C. J. McFall S.. (2021). Longitudinal Analysis of SARS-CoV-2 Vaccine Breakthrough Infections Reveal Limited Infectious Virus Shedding and Restricted Tissue Distribution. medRxiv 2021.08.30.21262701. doi: 10.1101/2021.08.30.21262701 PMC9047214 35791353

[B35] Liu Z. VanBlargan L. A. Bloyet L. M. Rothlauf P. W. Chen R. E. Stumpf S.. (2021). Identification of SARS-CoV-2 Spike Mutations That Attenuate Monoclonal and Serum Antibody Neutralization. Cell Host Microbe 29, 477–488.e4. doi: 10.1016/j.chom.2021.01.014 33535027 PMC7839837

[B6] Liu Y. Yan L. M. Wan L. Xiang T. X. Le A. Liu J. M.. (2020). Viral Dynamics in Mild and Severe Cases of COVID-19. Lancet Infect. Dis. 20, 656–657. doi: 10.1016/S1473-3099(20)30232-2 32199493 PMC7158902

[B27] Luo C. H. Morris C. P. Sachithanandham J. Amadi A. Gaston D. Li M.. (2021). Infection With the SARS-CoV-2 Delta Variant Is Associated With Higher Infectious Virus Loads Compared to the Alpha Variant in Both Unvaccinated and Vaccinated Individuals. medRxiv ciab986. doi: 10.1101/2021.08.15.21262077 PMC8903351 34922338

[B33] McCarthy K. R. Rennick L. J. Nambulli S. Robinson-McCarthy L. R. Bain W. G. Haidar G.. (2021). Recurrent Deletions in the SARS-CoV-2 Spike Glycoprotein Drive Antibody Escape. Science 371, 1139–1142. doi: 10.1126/science.abf6950 33536258 PMC7971772

[B22] Morris C. P. Luo C. H. Amadi A. Schwartz M. Gallagher N. Ray S. C.. (2021). An Update on SARS-CoV-2 Diversity in the United States National Capital Region: Evolution of Novel and Variants of Concern. Clin. Infect. Dis. ciab636. doi: 10.1093/cid/ciab636 34272947 PMC8406876

[B19] Mostafa H. H. Carroll K. C. Hicken R. Berry G. J. Manji R. Smith E.. (2020c). Multi-Center Evaluation of the Cepheid Xpert(R) Xpress SARS-CoV-2/Flu/RSV Test. J. Clin. Microbiol. 59 (3). doi: 10.1128/JCM.02955-20 PMC8106732 33298613

[B17] Mostafa H. H. Hardick J. Morehead E. Miller J. A. Gaydos C. A. Manabe Y. C. (2020a). Comparison of the Analytical Sensitivity of Seven Commonly Used Commercial SARS-CoV-2 Automated Molecular Assays. J. Clin. Virol. 130, 104578. doi: 10.1016/j.jcv.2020.104578 32777761 PMC7405824

[B18] Mostafa H. H. Lamson D. M. Uhteg K. Geahr M. Gluck L. de Cardenas J. N. B.. (2020b). Multicenter Evaluation of the NeuMoDx SARS-CoV-2 Test. J. Clin. Virol. 130, 104583. doi: 10.1016/j.jcv.2020.104583 32791382 PMC7413157

[B37] Mostafa H. H. Luo C. H. Morris C. P. Li M. Swanson N. J. Amadi A.. (2021). SARS-CoV-2 Infections in mRNA Vaccinated Individuals Are Biased for Viruses Encoding Spike E484K and Associated With Reduced Infectious Virus Loads That Correlate With Respiratory Antiviral IgG Levels. medRxiv 135, 104737. doi: 10.1016/j.jcv.2021.104737 PMC8979609 35398602

[B39] Niesen M. J. M. Anand P. Silvert E. Suratekar R. Pawlowski C. Ghosh P.. (2021). COVID-19 Vaccines Dampen Genomic Diversity of SARS-CoV-2: Unvaccinated Patients Exhibit More Antigenic Mutational Variance. medRxiv doi: 10.1101/2021.07.01.21259833

[B28] Planas D. Veyer D. Baidaliuk A. Staropoli I. Guivel-Benhassine F. Rajah M. M.. (2021). Reduced Sensitivity of SARS-CoV-2 Variant Delta to Antibody Neutralization. Nature 596, 276–280. doi: 10.1038/s41586-021-03777-9 34237773

[B24] Sethuraman N. Jeremiah S. S. Ryo A. (2020). Interpreting Diagnostic Tests for SARS-CoV-2. JAMA 323, 2249–2251. doi: 10.1001/jama.2020.8259 32374370

[B34] Suryadevara N. Shrihari S. Gilchuk P. VanBlargan L. A. Binshtein E. Zost S. J.. (2021). Neutralizing and Protective Human Monoclonal Antibodies Recognizing the N-Terminal Domain of the SARS-CoV-2 Spike Protein. Cell 184, 2316–2331.e15. doi: 10.1016/j.cell.2021.03.029 33773105 PMC7962591

[B11] Tarhini H. Recoing A. Bridier-Nahmias A. Rahi M. Lambert C. Martres P.. (2021). Long-Term Severe Acute Respiratory Syndrome Coronavirus 2 (SARS-CoV-2) Infectiousness Among Three Immunocompromised Patients: From Prolonged Viral Shedding to SARS-CoV-2 Superinfection. J. Infect. Dis. 223, 1522–1527. doi: 10.1093/infdis/jiab075 33556961 PMC7928754

[B15] Thielen P. M. Wohl S. Mehoke T. Ramakrishnan S. Kirsche M. Falade-Nwulia O.. (2021). Genomic Diversity of SARS-CoV-2 During Early Introduction Into the Baltimore-Washington Metropolitan Area. JCI Insight 6 (6), e144350. doi: 10.1172/jci.insight.144350 PMC8026189 33749660

[B13] Truong T. T. Ryutov A. Pandey U. Yee R. Goldberg L. Bhojwani D.. (2021). Increased Viral Variants in Children and Young Adults With Impaired Humoral Immunity and Persistent SARS-CoV-2 Infection: A Consecutive Case Series. EBioMedicine 67, 103355. doi: 10.1016/j.ebiom.2021.103355 33915337 PMC8072072

[B16] Uhteg K. Jarrett J. Richards M. Howard C. Morehead E. Geahr M.. (2020). Comparing the Analytical Performance of Three SARS-CoV-2 Molecular Diagnostic Assays. J. Clin. Virol. 127, 104384. doi: 10.1016/j.jcv.2020.104384 32361285 PMC7194987

[B23] Waskom M. L. (2021). Seaborn: Statistical Data Visualization. J. Open Source Softw. 6 (60), 3021. doi: 10.21105/joss.03021

[B31] Weisblum Y. Schmidt F. Zhang F. DaSilva J. Poston D. Lorenzi J. C.. (2020). Escape From Neutralizing Antibodies by SARS-CoV-2 Spike Protein Variants. Elife 9, 2020.07.21.214759. doi: 10.7554/eLife.61312.sa2 PMC7723407 33112236

[B7] Xu K. Chen Y. Yuan J. Yi P. Ding C. Wu W.. (2020). Factors Associated With Prolonged Viral RNA Shedding in Patients With Coronavirus Disease 2019 (COVID-19). Clin. Infect. Dis. 71, 799–806. doi: 10.1093/cid/ciaa351 32271376 PMC7184421

[B5] Zheng S. Fan J. Yu F. Feng B. Lou B. Zou Q.. (2020). Viral Load Dynamics and Disease Severity in Patients Infected With SARS-CoV-2 in Zhejiang Province, China, January-March 2020: Retrospective Cohort Study. BMJ 369, m1443. doi: 10.1136/bmj.m1443 32317267 PMC7190077

